# Mental health and psychological resilience amid the spread of the Omicron variant: a comparison between China and Korea

**DOI:** 10.3389/fpubh.2024.1451318

**Published:** 2025-01-07

**Authors:** Chenyuan Qin, Minjung Lee, Jie Deng, Yubin Lee, Myoungsoon You, Jue Liu

**Affiliations:** ^1^School of Public Health, Peking University, Beijing, China; ^2^Dental Research Institute, School of Dentistry, Seoul National University, Seoul, Republic of Korea; ^3^Department of Public Health Sciences, Graduate School of Public Health, Seoul National University, Seoul, Republic of Korea; ^4^Institute for Global Health and Development, Peking University, Beijing, China; ^5^National Health Commission Key Laboratory of Reproductive Health, Peking University, Beijing, China

**Keywords:** COVID-19, mental health, anxiety, depression, psychological resilience

## Abstract

**Objectives:**

Besides physical health risks, large public health events also exert negative impacts on people's mental health. We aimed to explore the prevalence and correlates of mental distress and its association with psychological resilience among countries amid the Omicron wave.

**Methods:**

We conducted cross-sectional surveys simultaneously in China and South Korea from March 15 to 30, 2023. Brief Resilience Scale (BRS), Generalized Anxiety Disorder 7-item (GAD-7) scale, and Patient Health Questionnaire-9 (PHQ-9) scale were used to measured psychological resilience and mental distress.

**Results:**

Self-reported rates of anxiety and depressive symptoms in 3,000 Chinese participants were 24.5% and 30.5%, while the above-mentioned rates were 17.2%and 34.4% in 1,000 Korean participants. Chinese participants had a marginally higher BRS score. Psychological resilience was inversely associated with the prevalence of anxiety symptoms and depressive symptoms. Similar results can be observed in Korea. Results remained robust in all models.

**Conclusion:**

Chinese and Korean populations reported a high prevalence of mental distress with variations in different characteristics, indicating practical implications for developing tailored mental health policies and services in the context of large public health events.

## Introduction

Coronavirus disease 2019 (COVID-19), a global public health emergency lasting for over 3 years, has inflicted tremendous challenges and threats to human life, health, and socioeconomic development (1–4). Besides physical health risks, the COVID-19 pandemic also exerted profound negative impacts on people's mental health (5–8). Studies have shown that during the COVID-19 pandemic, people have experienced various psychological problems, such as anxiety, depression, post-traumatic stress disorder (PTSD), and insomnia (8–12). These psychological problems not only affect individual well-being and quality of life but also may lead to serious consequences, such as social dysfunction, work impairment, and suicide risk (8–13).

Several studies have reported the prevalence of anxiety, depression, or other mental health problems among populations in different countries before or after the COVID-19 pandemic (8, 10, 11, 14–18). A systematic review and meta-analysis compared the general mental health, anxiety symptoms, and depressive symptoms of 134 cohorts before and after the COVID-19 pandemic and found no evidence in the general population of changes in general mental health, except for a slight deterioration in symptoms of depression (15). However, another systematic review found that due to the COVID-19 pandemic, the global prevalence of anxiety disorder and major depressive disorder increased by 25.6% and 27.6% during the first pandemic year, respectively (16). In the above study, the prevalence of anxiety disorder and major depressive disorder increased by 11.2% and 8.9% in China and increased by 11.3% and 10.4% in South Korea (16). Bareeqa et al. pooled the prevalence of depression in China in the early stage of the COVID-19 pandemic and found that the prevalence of depression was 26.9% (ranging from 5.9% to 50.7%), and the prevalence of anxiety was 21.8% (ranging from 6.1% to 44.7%; 17). According to a scientific quota survey design, South Korea conducted a mental health survey among national adults in March 2020 (18). They found that 19% of people had anxiety symptoms and 17.5% had some form of depression, much higher than the prevalence of depression in 2018 (2.8%) and anxiety in 2016 (5.7%; 18). Other subsequent studies, both in China and South Korea, have confirmed the severity of psychological problems during the pandemic (5, 6, 19). However, there is no study comparing the mental health status of Chinese and Korean populations 3 years into the COVID-19 pandemic, especially during the Omicron variant wave.

Psychological resilience refers to the ability of individuals to adapt and recover when facing stress or adversity (20). Psychological resilience is considered a protective factor that can mitigate the adverse effects of stress on mental health and promote individual psychological adaptation and growth (20–22). In extreme stress situations such as the COVID-19 pandemic, psychological resilience may play an important role in individual mental health (21). However, there is scant research on the role and influencing factors of psychological resilience in the context of the COVID-19 pandemic, especially cross-cultural comparative studies.

China and South Korea are among the countries in Asia that have been severely affected by the COVID-19 pandemic, and they are also countries with different cultural characteristics and social backgrounds (3). We adopted a cross-cultural perspective to explore the levels of psychological resilience and mental distress among Chinese and Korean populations amid the spread of the Omicron variant and compared their differences and connections. This study will provide evidence for further understanding the impact of the COVID-19 pandemic on the mental health of populations with different cultural backgrounds, offer a reference for developing targeted mental health policies and services, and guide the design of effective psychological resilience training and promotion strategies to cope with psychological stress.

## Methods

### Study design and participants

We conducted population-based cross-sectional surveys simultaneously in China and South Korea from March 15 to March 30, 2023, amid the spread of the SARS-CoV-2 Omicron variant. *SurveyStar* and *Korea Research*, two professional paid scientific data research companies, facilitated online platforms and diverse sample pools. Electronic questionnaires were randomly disseminated to potential participants who fulfilled the preplanned inclusion criteria based on total population distribution (geographical region, gender, and age) of both countries. Adults aged 18 and above from China/Korea were the potential subjects. Respondents consented to allow researchers to utilize anonymous data for academic purposes by completing and submitting the questionnaire.

We estimated the target sample size using PASS software 15.0 (NCSS LLC., Kaysville, U.T., USA) based on previous studies on the prevalence of anxiety and depression in China and South Korea during the COVID-19 pandemic, with a margin of error of ±5% and a confidence level of 95%. The minimum sample size for this study were 673 Chinese subjects and 527 Korean subjects (17, 18). We collected 3,025 initial questionnaires from China and 1,009 from Korea. After quality control and manual inspection, 3,000 Chinese participants and 1,000 Korean participants were included in the final analysis.

This study met the requirements of the Declaration of Helsinki and was approved by the Ethics Committee of Peking University (IRB00001052-21126).

### Procedures

#### Mental distress

We measured generalized anxiety and depressive symptoms as indicators of mental distress among participants in this study. The Generalized Anxiety Disorder 7-item (GAD-7) scale is a self-report instrument comprising seven items that correspond to the respondents' self-perceived anxiety in the past 2 weeks (23). Each item entails four scoring levels (0 = not at all; 1 = occasionally; 2 = more than half the time; 3 = nearly every day), with a total score range of 0–21. To evaluate self-reported depressive symptoms and their severity, we used the Patient Health Questionnaire-9 (PHQ-9) scale, which is the most prevalent depression screening tool in primary health care (24). Respondents were instructed to self-rate themselves on four progressive levels (0 = not at all; 1 = occasionally; 2 = more than half the time; 3 = nearly every day) based on their situation in the past 2 weeks. The PHQ-9 scale has a scoring range of 0–27. Higher scores signify higher symptom burden.

Both scales have been extensively validated and reliable in cross-cultural settings (6, 23–26). Previous studies have indicated that a score of 10 is more clinically relevant as a cutoff point for anxiety and depressive symptoms (27, 28). Therefore, we defined GAD-7 ≥ 10 as positive for anxiety symptoms and PHQ-9 ≥ 10 as positive for depressive symptoms in this study.

#### Psychological resilience

Psychological resilience was measured by the Brief Resilience Scale (BRS), which consists of six items and assesses the individual's perceived ability to preserve and restore health after illness or loss (e.g., I tend to bounce back rapidly from stressful events; 29). The scale adopts a 5-point Likert scoring scheme (1 = strongly disagree, 2 = disagree, 3 = neutral, 4 = agree, 5 = strongly agree), with half of the items scored positively and the other half scored negatively (29). The average score of the six items is the BRS score, ranging from 1 to 5, with higher scores indicating higher psychological resilience. To facilitate statistical analysis, we stratified all eligible respondents into three groups according to their scores: low (< 3 points), moderate (3.0–4.3 points), and high [>4.3 points; (29, 30)]. The BRS has been cross-culturally adapted and applied in numerous countries and has demonstrated good reliability and validity (21, 29, 31).

#### Covariates

Sociodemographic characteristics and health-related factors were collected as the main covariates in this study. The sociodemographic characteristics encompassed age, gender, location, marital status, income level, household size, housing, and children. Chronic disease status was assessed by posing the question “Have you ever received a diagnosis of any chronic diseases: hypertension, diabetes, dyslipidemia, tuberculosis, asthma, osteoarthritis, rheumatoid arthritis, etc.?” Moreover, we specifically probed the participants' COVID-19 infection history as a key covariate.

### Statistical analysis

Statistical descriptions of the basic characteristics, prevalence of anxiety symptoms, prevalence of depressive symptoms, and psychological resilience scores of the study participants were conducted. Continuous variables were described by the mean (M) and standard deviation (SD) and compared by *t*-test or analysis of variance (ANOVA). Categorical variables were described by frequency and percentage and compared across groups by the chi-square test or Fisher's exact test. Modified Poisson regression models with log link function and robust standard error were employed to examine the association between psychological resilience and mental distress indicators. A series of potential confounding factors were adjusted for in different models to assess the robustness of the estimations, reported by crude relative risks (cRRs) and adjusted relative risks (aRRs) with 95% CIs. Model A was a univariable model. Model B was adjusted for sociodemographic characteristics (age, gender, location, education, marital status, household size, housing, income, and children), and then we added sociodemographic characteristics and chronic disease history in Model C. COVID-19 history was additionally controlled in Model D. Furthermore, we explored the interactions of psychological resilience with covariates and conducted subgroup analyses based on stratified sampling to determine the associations of mental distress with psychological resilience across different levels of attributes.

We used SPSS 26.0 (IBM SPSS Inc., New York, USA) to conduct statistical analyses and set the significance level at a two-sided *P*-value of < 0.05.

## Results

### Sociodemographic and health-related characteristics

A total of 3,000 participants from China and 1,000 participants from Korea were finally recruited for this study. The frequencies and percentages of sociodemographic and other key variables are presented in Table 1. Chinese respondents were predominantly under 40 years old (88.6%), whereas over half of the Korean respondents were at least 50 years old, which partly accounted for the higher education level of Chinese respondents. Among Chinese participants, 40.8% were male, 65.2% lived in urban areas, 73.4% were married, and most of them had a household size of three or more (82.4%). In contrast, 49.5% of Korean respondents were male, 85.35% lived in urban areas, and 56.7% were married but had a slightly lower proportion of household size of three or more (55.3%) than China. Moreover, 89.7% of Chinese respondents reported a history of COVID-19 infection, while the previous infection rate was somewhat lower (61.4%) among Korean respondents.

**Table 1:** Basic characteristics of participants in China and Korea.

**Characteristics**	**China (*N* = 3,000)**	**Korea (*N* = 1,000)**	***P*-value**
	***n*** **(%)**	***n*** **(%)**	
**Sex**			< 0.001^*^
Male	1,225 (40.8)	495 (49.5)	
Female	1,775 (59.2)	505 (50.5)	
**Age (years)**			< 0.001^*^
< 30	1,385 (46.2)	166 (16.6)	
30–39	1,271 (42.4)	149 (14.9)	
40–49	249 (8.3)	183 (18.3)	
≥50	95 (3.2)	502 (50.2)	
**Location**			< 0.001^*^
Urban	1,957 (65.2)	853 (85.3)	
Rural	1,043 (34.8)	147 (14.7)	
**Education** ^†^			< 0.001^*^
High school and below	552 (18.4)	573 (57.3)	
Bachelor degree	2,226 (74.2)	387 (38.7)	
Master degree	222 (7.4)	40 (4.0)	
**Marital status**			< 0.001^*^
Unmarried	774 (25.8)	327 (32.7)	
Married	2,203 (73.4)	567 (56.7)	
Others	23 (0.8)	106 (10.6)	
**Household size**			< 0.001^*^
Solitary	227 (7.6)	160 (16.0)	
2	299 (10.0)	287 (28.7)	
3	1,317 (43.9)	263 (26.3)	
≥4	1,157 (38.6)	290 (29.0)	
**Housing**			< 0.001^*^
Purchased	2,465 (82.2)	636 (63.6)	
Rental	535 (17.8)	364 (36.4)	
**Income** ^§^			< 0.001^*^
Lower level	193 (6.4)	351 (35.1)	
Higher level	2,807 (93.6)	649 (64.9)	
**Young children** ^‡^			< 0.001^*^
No	1,115 (37.2)	677 (67.7)	
Yes	1,885 (62.8)	323 (32.3)	
**Chronic disease**			< 0.001^*^
No	2,160 (72.0)	586 (58.6)	
Yes	840 (28.0)	414 (41.4)	
**COVID-19 history**			< 0.001^*^
No	310 (10.3)	386 (38.6)	
Yes	2,690 (89.7)	614 (61.4)	

^*^A P < 0.05 is considered to be statistically significant.

^†^Junior college in China was assigned to “high school and below” in our analyses.

^§^We divided the income brackets based on the official average monthly income for China and Korea in 2022. Combining our questionnaire setup, we set income below 3,000 RMB (China) and 3 million won (Korea) as the “Lower level.”

^‡^Young children were defined as children attending high school or younger.

### Prevalence of anxiety symptoms and depressive symptoms in China and Korea

Table 2 shows the self-reported rates of anxiety and depressive symptoms in China and Korea amid the spread of the Omicron variant. Self-reported rates of anxiety symptoms and depressive symptoms in China were 24.5% (95% CI: 23.0–26.1) and 30.5% (95% CI: 28.8–32.1), while the anxiety rate in Korea was lower than that in China (17.2% vs. 24.5%), and the depression rate was relatively higher (34.4% vs. 30.5%). For mental distress (positive for either anxiety or depressive symptoms), the self-reported positive rates were comparable between China and Korea (35.5% vs. 35.7%).

**Table 2:** Prevalence of anxiety and depressive symptoms in China and Korea amid the spread of Omicron variant.

**Country**	**Characteristics**	**Anxiety symptom**			**Depressive symptom**			**Any of the two**		
		***n*** **(%)**	**95% CI**	***P*** **-value**	***n*** **(%)**	**95% CI**	***P*** **-value**	***n*** **(%)**	**95% CI**	***P*** **-value**
**China (*****N*** **=** **3,000)**	**Total**	735 (24.5)	23.0–26.1		914 (30.5)	28.8–32.1		1,065 (35.5)	33.8–37.2	
	**Sex**			0.068			0.142			0.105
	Male	279 (22.8)	20.5–25.2		355 (29.0)	26.5–31.6		414 (33.8)	31.2–36.5	
	Female	456 (25.7)	23.7–27.8		559 (31.5)	29.4–33.7		651 (36.7)	34.5–38.9	
	**Age (years)**			< 0.001^*^			< 0.001^*^			< 0.001^*^
	< 30	416 (30.0)	27.7–32.5		503 (36.3)	33.8–38.9		587 (42.4)	39.8–45.0	
	30–39	266 (20.9)	18.8–23.2		346 (27.2)	24.8–29.7		399 (31.4)	28.9–34.0	
	40–49	39 (15.7)	11.6–20.6		45 (18.1)	13.7–23.2		57 (22.9)	18.0–28.4	
	≥50	14 (14.7)	8.7–22.9		20 (21.1)	13.8–30.0		22 (23.2)	15.6–32.4	
	**Location**			< 0.001^*^			< 0.001^*^			< 0.001^*^
	Urban	403 (20.6)	18.8–22.4		510 (26.1)	24.2–26.1		603 (30.8)	28.8–32.9	
	Rural	332 (31.8)	29.1–34.7		404 (38.7)	35.8–38.7		462 (44.3)	41.3–47.3	
	**Education** ^†^			0.01^*^			0.001^*^			< 0.001^*^
	High school and below	162 (29.3)	25.7–33.2		203 (36.8)	32.8–40.9		241 (43.7)	39.6–47.8	
	Bachelor degree	516 (23.2)	21.5–25.0		654 (29.4)	27.5–31.3		748 (33.6)	31.7–35.6	
	Master degree	57 (25.7)	20.3–31.7		57 (25.7)	20.3–31.7		76 (34.2)	28.2–40.6	
	**Marital status**			< 0.001^*^			< 0.001^*^			< 0.001^*^
	Unmarried	251 (32.4)	29.2–35.8		299 (38.6)	35.2–42.1		346 (44.7)	41.2–48.2	
	Married	476 (21.6)	19.9–23.4		603 (27.4)	25.5–29.3		705 (32.0)	30.1–34.0	
	Others	8 (34.8)	18.0–55.1		12 (52.2)	32.5–71.3		14 (60.9)	40.6–78.6	
	**Household size**			< 0.001^*^			0.001^*^			< 0.001^*^
	Solitary	80 (28.8)	29.2–41.6		92 (35.5)	34.3–47.0		105 (42.1)	39.9–52.8	
	2	86 (22.3)	23.9–34.1		106 (28.6)	30.2–41.0		126 (33.3)	36.6–47.8	
	3	294 (23.8)	20.1–24.6		377 (29.3)	26.2–31.1		438 (34.2)	30.8–35.8	
	≥4	275 (22.6)	21.4–26.3		339 (28.4)	26.7–32.0		396 (33.1)	31.5–37.0	
	**Housing**			< 0.001^*^			< 0.001^*^			< 0.001^*^
	Purchased	557 (22.6)	21.0–24.3		701 (28.4)	26.7–30.2		816 (33.1)	31.3–35.0	
	Rental	178 (33.3)	29.4–37.3		213 (39.8)	35.7–44.0		249 (46.5)	42.3–50.8	
	**Income** ^**§**^			< 0.001^*^			< 0.001^*^			< 0.001^*^
	Lower level	72 (37.3)	30.7–44.3		88 (45.6)	38.7–52.6		99 (51.3)	44.3–58.3	
	Higher level	663 (23.6)	22.1–25.2		826 (29.4)	27.8–31.1		966 (34.4)	32.7–36.2	
	**Young children** ^‡^			< 0.001^*^			< 0.001^*^			< 0.001^*^
	No	335 (30.0)	27.4–32.8		396 (35.5)	32.7–38.4		466 (41.8)	38.9–44.7	
	Yes	400 (21.2)	19.4–23.1		518 (27.5)	25.5–29.5		599 (31.8)	29.7–33.9	
	**COVID-19 history**			0.096			0.653			0.313
	No	64 (20.6)	16.4–25.4		91 (29.4)	24.5–34.6		102 (32.9)	27.9–38.3	
	Yes	671 (24.9)	23.3–26.6		823 (30.6)	28.9–32.4		963 (35.8)	34.0–37.6	
	**Chronic disease**			< 0.001^*^			< 0.001^*^			< 0.001^*^
	No	471 (21.8)	20.1–23.6		590 (27.3)	25.5–29.2		697 (32.3)	30.3–34.3	
	Yes	264 (31.4)	28.4–34.6		324 (38.6)	35.3–41.9		368 (43.8)	40.5–47.2	
	**Psychological resilience**			< 0.001^*^			< 0.001^*^			< 0.001^*^
	Low	197 (64.0)	58.5–69.2		215 (69.8)	64.5–74.7		240 (77.9)	73.0–82.3	
	Moderate	489 (26.9)	24.9–28.9		651 (35.7)	33.6–38.0		752 (41.3)	39.0–43.6	
	High	49 (5.6)	4.2–7.3		48 (5.5)	4.1–7.2		73 (8.4)	6.7–10.4	
**Korea (*****N*** **=** **1,000)**	**Total**	172 (17.2)	15.0–19.6		344 (34.4)	31.5–37.4		357 (35.7)	32.8–38.7	
	**Sex**			0.138			0.865			0.624
	Male	94 (19.0)	15.7–22.6		169 (34.1)	30.1–38.4		173 (34.9)	30.8–39.2	
	Female	78 (15.4)	12.5–18.8		175 (34.7)	30.6–38.9		184 (36.4)	32.3–40.7	
	**Age (years)**			< 0.001^*^			0.134			0.063
	< 30	36 (21.7)	15.9–28.4		62 (37.3)	30.3–44.9		65 (39.2)	32.0–46.7	
	30–39	36 (24.2)	17.8–31.5		57 (38.3)	30.7–46.2		58 (38.9)	31.4–46.9	
	40–49	41 (22.4)	16.8–28.8		70 (38.3)	31.4–45.4		75 (41.0)	34.0–48.2	
	≥50	59 (11.8)	9.2–14.8		155 (30.9)	27.0–35.0		159 (31.7)	27.7–35.8	
	**Location**			0.264			0.768			0.644
	Urban	142 (16.6)	14.3–19.3		295 (34.6)	31.4–37.8		307 (36.0)	32.8–39.3	
	Rural	30 (20.4)	14.5–27.5		49 (33.3)	26.1–41.2		50 (34.0)	26.7–41.9	
	**Education** ^†^			0.468			0.122			0.142
	High school and below	100 (17.5)	14.5–20.7		205 (35.8)	31.9–39.8		214 (37.3)	33.5–41.4	
	Bachelor degree	68 (17.6)	14.0–21.6		131 (33.9)	29.3–38.7		134 (34.6)	30.0–39.5	
	Master degree	4 (10.0)	3.5–22.0		8 (20.0)	9.9–34.2		9 (22.5)	11.8–37.1	
	**Marital status**			0.004^*^			0.011^*^			0.003^*^
	Unmarried	75 (22.9)	18.6–27.7		131 (40.1)	34.9–45.4		139 (42.5)	37.2–47.9	
	Married	81 (14.3)	11.6–17.3		173 (30.5)	26.8–34.4		178 (31.4)	27.7–35.3	
	Others	16 (15.1)	9.3–22.8		40 (37.7)	28.9–47.2		40 (37.7)	28.9–47.2	
	**Household size**			0.071			0.028^*^			0.018^*^
	Solitary	37 (23.1)	17.1–30.1		67 (41.9)	34.4–49.6		70 (43.8)	36.2–51.5	
	2	52 (18.1)	14.0–22.9		103 (35.9)	30.5–41.6		107 (37.3)	31.8–43.0	
	3	44 (16.7)	12.6–21.6		92 (35.0)	29.4–40.9		95 (36.1)	30.5–42.1	
	≥4	39 (13.4)	9.9–17.7		82 (28.3)	23.3–33.7		85 (29.3)	24.3–34.7	
	**Housing**			0.07			< 0.001^*^			< 0.001^*^
	Purchased	99 (15.6)	12.9–18.5		190 (29.9)	26.4–33.5		200 (31.4)	27.9–35.1	
	Rental	73 (20.1)	16.2–24.4		154 (42.3)	37.3–47.4		157 (43.1)	38.1–48.3	
	**Income** ^**§**^			0.001^*^			< 0.001^*^			< 0.001^*^
	Lower level	80 (22.8)	18.6–27.4		147 (41.9)	36.8–47.1		152 (43.3)	38.2–48.5	
	Higher level	92 (14.2)	11.7–17.0		197 (30.4)	26.9–34.0		205 (31.6)	28.1–35.2	
	**Young children** ^‡^			0.796			0.874			0.543
	No	115 (17.0)	14.3–20.0		234 (34.6)	31.1–38.2		246 (36.3)	32.8–40.0	
	Yes	72 (18.7)	15.0–22.8		110 (34.1)	29.0–39.3		111 (34.4)	29.3–39.7	
	**COVID-19 history**			0.334			0.324			0.266
	No	72 (18.7)	15.0–22.8		140 (36.3)	31.6–41.2		146 (37.8)	33.1–42.7	
	Yes	100 (16.3)	13.5–19.4		204 (33.2)	29.6–37.0		211 (34.4)	30.7–38.2	
	**Chronic disease**			0.019^*^			0.005^*^			0.030^*^
	No	87 (14.8)	12.1–17.9		181 (30.9)	27.2–34.7		193 (32.9)	29.2–36.8	
	Yes	85 (20.5)	16.9–24.6		163 (39.4)	34.8–44.1		164 (39.6)	35.0–44.4	
	**Psychological resilience**			< 0.001^*^			< 0.001^*^			< 0.001^*^
	Low	71 (37.0)	30.4–44.0		119 (62.0)	55.0–68.6		122 (63.5)	56.6–70.1	
	Moderate	94 (14.7)	12.1–17.6		212 (33.2)	29.6–36.9		220 (34.4)	30.8–38.2	
	High	7 (4.1)	1.9–8.0		13 (7.7)	4.4–12.4		15 (8.9)	5.3–13.9	

^*^A P < 0.05 is considered to be statistically significant.

^†^Junior college in China was assigned to “high school and below” in our analyses.

^§^We divided the income brackets based on the official average monthly income for China and Korea in 2022. Combining our questionnaire setup, we set income below 3,000 RMB (China) and 3 million won (Korea) as the “Lower level.”

^‡^Young children were defined as children attending high school or younger.

Among 3,000 Chinese participants, people with the following characteristics had a significantly higher self-reported rate of anxiety symptoms (Table 2): younger age, rural residence, lower education level, unmarried status, living alone or in a rental a house, lower income, having no young children, having chronic diseases, and lower psychological resilience. The distribution of depressive symptoms and any mental distress with different characteristics followed a similar pattern as that of anxiety symptoms. In Korea, both anxiety and depressive symptoms were more prevalent among participants who were unmarried, low-income, chronically ill, or less resilient (Table 2). In addition, anxiety symptoms were more common among younger participants, whereas depressive symptoms were more frequent among those who lived alone or rented a house (Table 2).

As shown in Table 2 and Supplementary Tables 1, 2, for Chinese participants with a history of COVID-19 infection (*n* = 2,690), the rates of positive anxiety symptoms and depressive symptoms were 24.9% (95% CI: 23.3–26.6) and 30.6% (95% CI: 28.9–32.4), respectively, whereas the prevalence of self-reported anxiety and depression in Korea (*n* = 614) were 16.3% (95% CI: 13.5–19.4) and 33.2% (95% CI: 29.6–37.0). For those without a history of COVID-19 infection, positive rates of anxiety and depressive symptoms were reported as 20.9% (95% CI: 16.4–25.4) and 29.4% (95% CI: 24.5–34.6) of Chinese participants (*n* = 310), while the two self-reported rates in Korea (*n* = 386) were 18.7% (95% CI: 15.0–22.8) and 36.3% (95% CI: 31.6-41.2). In addition, we also display the distribution of mental distress by different characteristics among Chinese and South Korean participants with or without COVID-19 history in Supplementary Tables 1, 2.

### Psychological resilience of participants in China and Korea

Table 3 presents the Brief Resilience Scale (BRS) scores of Chinese and Korean participants in total and different subgroups of characteristics, showing their conditional psychological resilience. Our results indicated that Chinese participants had a BRS score of 3.34 ± 0.68, while the score was marginally lower in Korea (3.04 ± 0.71). Participants with self-reported anxiety symptoms and depressive symptoms tended to have higher BRS scores. Higher BRS scores, reflecting a stronger ability to bounce back or recover from adversity, were significantly related to older age, urban location, higher education level, married status, larger family size, purchased housing, higher income level, having young children, and absence of chronic diseases in China. Subgroups of marital status, housing, income and chronic diseases showed significant differences in BRS scores among Korean participants, while other subgroups did not.

**Table 3:** Psychological resilience in China and Korea amid the spread of Omicron variant.

**Characteristic**	**China**						**South Korea**					
	**All participants (*****N*** = **3,000)**		**Participants with COVID-19 history (*****N*** = **2,690)**		**Participants without COVID-19 history (*****N*** = **310)**		**All participants (*****N*** = **1,000)**		**Participants with COVID-19 history (*****N*** = **614)**		**Participants without COVID-19 history (*****N*** = **386)**	
	**M** ±**SD**	***P*** **-value**	**M** ±**SD**	***P*** **-value**	**M** ±**SD**	***P*** **-value**	**M** ±**SD**	***P*** **-value**	**M** ±**SD**	***P*** **-value**	**M** ±**SD**	***P*** **-value**
**Total**	3.34 ± 0.68		3.34 ± 0.67		3.36 ± 0.71		3.04 ± 0.71		3.07 ± 0.69		3.00 ± 0.74	
**Sex**		< 0.001^*^		< 0.001^*^		0.028^*^		0.763		0.403		0.727
Male	3.42 ± 0.68		3.42 ± 0.67		3.44 ± 0.70		3.05 ± 0.70		3.10 ± 0.69		2.98 ± 0.69	
Female	3.28 ± 0.67		3.29 ± 0.67		3.27 ± 0.71		3.04 ± 0.73		3.05 ± 0.69		3.01 ± 0.79	
**Age (years)**		< 0.001^*^		< 0.001^*^		0.001^*^		0.213		0.342¶		0.078
< 30	3.24 ± 0.67		3.24 ± 0.66		3.20 ± 0.70		2.95 ± 0.80		3.03 ± 0.78		2.71 ± 0.80	
30–39	3.41 ± 0.68		3.40 ± 0.68		3.52 ± 0.71		3.00 ± 0.79		3.02 ± 0.79		2.96 ± 0.79	
40–49	3.49 ± 0.64		3.47 ± 0.65		3.60 ± 0.57		3.06 ± 0.68		3.07 ± 0.70		3.06 ± 0.65	
≥50	3.52 ± 0.63		3.57 ± 0.60		3.27 ± 0.68		3.08 ± 0.67		3.12 ± 0.60		3.03 ± 0.73	
**Location**		< 0.001^*^		< 0.001^*^		0.008^*^		0.803		0.994		0.614
Urban	3.42 ± 0.68		3.41 ± 0.67		3.44 ± 0.70		3.04 ± 0.71		3.07 ± 0.70		2.99 ± 0.73	
Rural	3.20 ± 0.65		3.20 ± 0.65		3.22 ± 0.70		3.06 ± 0.70		3.07 ± 0.64		3.04 ± 0.77	
**Education** ^†^		0.040^*^		0.158		0.077		0.462		0.440		0.806
High school and below	3.28 ± 0.66		3.29 ± 0.66		3.21 ± 0.68		3.02 ± 0.71		3.05 ± 0.65		2.98 ± 0.79	
Bachelor degree	3.35 ± 0.68		3.35 ± 0.67		3.38 ± 0.71		3.07 ± 0.72		3.09 ± 0.75		3.03 ± 0.65	
Master degree	3.39 ± 0.70		3.36 ± 0.69		3.56 ± 0.73		3.14 ± 0.65		3.22 ± 0.63		2.92 ± 0.66	
**Marital status**		< 0.001^*^		< 0.001^*^		0.001^*^		0.037^*^		0.182		0.152
Unmarried	3.14 ± 0.66		3.14 ± 0.66		3.18 ± 0.69		2.96 ± 0.77		3.00 ± 0.75		2.89 ± 0.79	
Married	3.41 ± 0.67		3.41 ± 0.66		3.46 ± 0.70		3.09 ± 0.67		3.12 ± 0.66		3.04 ± 0.69	
Others	3.12 ± 0.66		3.16 ± 0.67		2.67 ± 0.47		3.07 ± 0.72		3.06 ± 0.65		3.08 ± 0.79	
**Household size**		< 0.001^*^		< 0.001^*^		0.405		0.096		0.068		0.562
Solitary	3.16 ± 0.68		3.16 ± 0.65		3.17 ± 0.80		2.97 ± 0.75		2.92 ± 0.71		3.03 ± 0.80	
2	3.25 ± 0.69		3.23 ± 0.69		3.38 ± 0.70		3.01 ± 0.67		3.04 ± 0.63		2.97 ± 0.72	
3	3.36 ± 0.68		3.36 ± 0.67		3.37 ± 0.71		3.13 ± 0.71		3.16 ± 0.69		3.08 ± 0.76	
≥4	3.37 ± 0.67		3.37 ± 0.66		3.40 ± 0.68		3.04 ± 0.72		3.09 ± 0.73		2.93 ± 0.69	
**Housing**		< 0.001^*^		< 0.001^*^		0.007^*^		0.009^*^		0.084		0.041^*^
Purchased	3.39 ± 0.68		3.38 ± 0.67		3.41 ± 0.70		3.09 ± 0.70		3.11 ± 0.68		3.05 ± 0.74	
Rental	3.13 ± 0.64		3.13 ± 0.63		3.15 ± 0.70		2.97 ± 0.72		3.01 ± 0.72		2.89 ± 0.73	
**Income** ^**§**^		< 0.001^*^		< 0.001^*^		< 0.001^*^		0.001^*^		0.011^*^		0.085
Lower level	3.07 ± 0.62		3.09 ± 0.64		2.95 ± 0.46		2.95 ± 0.72		2.97 ± 0.68		2.92 ± 0.76	
Higher level	3.36 ± 0.68		3.36 ± 0.67		3.40 ± 0.71		3.10 ± 0.70		3.12 ± 0.69		3.05 ± 0.72	
**Young children** ^‡^		< 0.001^*^		< 0.001^*^		0.005^*^		0.349		0.956		0.214
No	3.21 ± 0.68		3.20 ± 0.67		3.22 ± 0.70		3.03 ± 0.72		3.07 ± 0.69		2.97 ± 0.75	
Yes	3.42 ± 0.66		3.41 ± 0.66		3.48 ± 0.69		3.07 ± 0.70		3.08 ± 0.71		3.07 ± 0.69	
**Chronic disease**		0.024^*^		0.012^*^		0.602		0.009^*^		0.371		0.005^*^
No	3.36 ± 0.68		3.36 ± 0.67		3.35 ± 0.71		3.09 ± 0.75		3.09 ± 0.75		3.09 ± 0.74	
Yes	3.3 ± 0.67		3.29 ± 0.67		3.40 ± 0.68		2.98 ± 0.65		3.04 ± 0.59		2.88 ± 0.72	
**Anxiety symptom**		< 0.001^*^		< 0.001^*^		< 0.001^*^		< 0.001^*^		< 0.001^*^		< 0.001^*^
No	3.50 ± 0.61		3.50 ± 0.61		3.50 ± 0.64		3.15 ± 0.67		3.17 ± 0.65		3.11 ± 0.70	
Yes	2.84 ± 0.62		2.84 ± 0.61		2.79 ± 0.68		2.55 ± 0.69		2.59 ± 0.69		2.50 ± 0.69	
**Depressive symptom**		< 0.001^*^		< 0.001^*^		< 0.001^*^		< 0.001^*^		< 0.001^*^		< 0.001^*^
No	3.55 ± 0.60		3.55 ± 0.60		3.57 ± 0.62		3.24 ± 0.66		3.25 ± 0.65		3.24 ± 0.67	
Yes	2.86 ± 0.59		2.86 ± 0.59		2.84 ± 0.63		2.66 ± 0.65		2.72 ± 0.64		2.57 ± 0.65	
**Any of the two**		< 0.001^*^		< 0.001^*^		< 0.001^*^		< 0.001^*^		< 0.001^*^		< 0.001^*^
No	3.59 ± 0.58		3.59 ± 0.58		3.58 ± 0.62		3.25 ± 0.65		3.26 ± 0.65		3.25 ± 0.67	
Yes	2.89 ± 0.60		2.89 ± 0.60		2.91 ± 0.65		2.67 ± 0.65		2.73 ± 0.65		2.58 ± 0.65	

^*^A P < 0.05 is considered to be statistically significant.

^†^Junior college in China was assigned to “high school and below” in our analyses.

^§^ We divided the income brackets based on the official average monthly income for China and Korea in 2022. Combining our questionnaire setup, we set income below 3,000 RMB (China) and 3 million won (Korea) as the “Lower level.”

¶ Fisher exact test.

^‡^Young children were defined as children attending high school or younger.

### Association between psychological resilience and mental distress

Table 4 displays the results of the associations between psychological resilience and mental distress in China and Korea, in which the high-resilience group was set as the reference group in all models. In the unadjusted model of Chinese participants, psychological resilience was inversely associated with the prevalence of self-reported anxiety symptoms (low: cRR = 22.49, 95% CI: 9.42–53.66; moderate: cRR = 7.75, 95% CI: 3.24–18.54), depressive symptoms (low: cRR = 13.83, 95% CI: 7.53–25.43; moderate: cRR = 4.84, 95% CI: 2.62–8.92) and any of the two adverse mental health outcomes (low: cRR = 12.98, 95% CI: 7.47–22.55; moderate: cRR = 4.88, 95% CI: 2.80–8.50). A similar relationship can be observed in Korea, with lower psychological resilience being significantly associated with an increased risk of anxiety symptoms (low: cRR = 10.81, 95% CI: 1.56–75.11) and depressive symptoms (low: cRR = 6.33, 95% CI: 2.13–18.78).

**Table 4:** Association between psychological resilience and mental distress in China and Korea amid the spread of Omicron variant.

**Models**	**China** ^**^		**Korea** ^**^	
	**Low resilience RR (95% CI)**	**Moderate resilience RR (95% CI)**	**Low resilience RR (95% CI)**	**Moderate resilience RR (95% CI)**
**Model A**				
Anxiety symptom	22.49 (9.42, 53.66)^*^	7.75 (3.24, 18.54)^*^	10.81 (1.56, 75.11)^*^	3.47 (0.49, 24.35)
Depressive symptom	13.83 (7.53, 25.43)^*^	4.84 (2.62, 8.92)^*^	6.33 (2.13, 18.78)^*^	2.87 (0.96, 8.57)
Any of the two	12.98 (7.47, 22.55)^*^	4.88 (2.80, 8.50)^*^	6.61 (2.23, 19.61)^*^	2.95 (0.99, 8.81)
**Model B**				
Anxiety symptom	19.72 (8.25, 47.15)^*^	7.24 (3.03, 17.32)^*^	10.45 (1.55, 70.67)^*^	3.60 (0.53, 24.46)
Depressive symptom	12.28 (6.68, 22.58)^*^	4.54 (2.47, 8.36)^*^	6.19 (2.08, 18.38)^*^	2.91 (0.97, 8.68)
Any of the two	11.60 (6.68, 20.17)^*^	4.61 (2.65, 8.02)^*^	6.83 (4.16, 11.20)^*^	3.75 (2.29, 6.12)^*^
**Model C**				
Anxiety symptom	18.23 (7.63, 43.57)^*^	6.89 (2.89, 16.47)^*^	8.92 (1.31, 60.66)^*^	3.13 (0.46, 21.35)
Depressive symptom	11.44 (6.22, 21.04)^*^	4.35 (2.36, 8.00)^*^	5.69 (1.93, 16.78)^*^	2.70 (0.91, 7.99)
Any of the two	10.89 (6.27, 18.93)^*^	4.43 (2.55, 7.71)^*^	6.08 (2.06, 17.97)^*^	2.84 (0.96, 8.43)
**Model D**				
Anxiety symptom	18.14 (7.59, 43.36)^*^	6.86 (2.87, 16.38)^*^	8.91 (1.32, 60.40)^*^	3.14 (0.46, 21.32)
Depressive symptom	11.44 (6.22, 21.04)^*^	4.34 (2.36, 8.00)^*^	5.69 (1.93, 16.78)^*^	2.70 (0.91, 7.99)
Any of the two	10.87 (6.25, 18.90)^*^	4.42 (2.54, 7.69)^*^	6.08 (2.06, 17.96)^*^	2.84 (0.96, 8.43)

^*^ A P < 0.05 is considered to be statistically significant.

^**^ The high resilience group were set as the reference group in all models.

Model A is a univariable modified Poisson regression model. Model B is adjusted by sociodemographic characteristics (age; gender; location; education; marital status; household size; housing; income; children). Model C is adjusted by sociodemographic characteristics and chronic disease history. Model D is adjusted by sociodemographic characteristics, chronic disease history, and COVID-19 history.

After adjusting for sociodemographic characteristics in model B and chronic diseases in model C, the negative association between psychological resilience and mental distress indicators remained robust and seemed to be more pronounced in China (Table 4). When controlling for all covariates in model D, Chinese participants with low and moderate psychological resilience had an 18.14-fold (95% CI: 7.59–43.36) and a 6.86-fold (95% CI: 2.87–16.38) higher risk of self-reported anxiety symptoms than those with high psychological resilience, respectively. The effect of lower psychological resilience was still evident in increasing the risk of depressive symptoms (low: aRR = 11.44, 95% CI: 6.22–21.04; moderate: aRR = 4.34, 95% CI: 2.36–8.00). The abovementioned inverse association between psychological resilience and mental distress indicators persisted among Korean participants, although it was more prominent among Chinese participants.

We also stratified the analysis by COVID-19 infection history and found robust significant associations (Supplementary Table 3).

### Subgroup analysis of the association between psychological resilience and mental distress

Supplementary Tables 4, 5 illustrate the results of subgroup analyses evaluating the association between psychological resilience and three mental distress indicators. In the subgroup analysis of Chinese participants (Supplementary Table 4), the associations between psychological resilience and anxiety symptoms or depressive symptoms were not modified by any covariates except for location and housing (all *P* for interaction >0.05). Conversely, age, location, housing, and chronic disease markedly modified the association between psychological resilience and any of the two mental distress categories (all *P* for interaction < 0.05), but the inverse relationship remained significant.

Among South Korean participants (Supplementary Table 5), the association between psychological resilience and all three mental distress indicators did not vary when stratified by any covariate (all *P* for interaction >0.05). Marital status and income level were the only two covariates that modified the association between psychological resilience and depressive symptoms (*P* for interaction < 0.05), and similar modifying effects can be seen in the interactional analyses of resilience and total mental distress.

## Discussion

This study explored the cross-cultural variations and associations among the mental health outcomes in China and Korea amid the Omicron variant surge, providing valuable data and insights for comparative analysis. Our results revealed that the prevalence of self-reported anxiety and depressive symptoms were 24.5% and 30.5% in China, and Korean participants had a lower self-reported rate of anxiety symptoms (17.2% vs. 24.5%) and a higher self-reported rate of depressive symptoms (34.4% vs. 30.5%). Chinese participants scored higher on psychological resilience than Korean participants. Significant negative associations between psychological resilience and mental distress indicators were stable across models adjusted by various covariates in both countries. Therefore, this study highlighted the importance of addressing mental health issues during the critical period of the Omicron variant outbreak and suggested enhancing psychological resilience as a potential strategy to reduce the harmful effects of mental distress.

The results of this survey indicate that both China and South Korea have a non-negligible prevalence of mental distress, suggesting that the general population may experience serious emotional distress during public health emergencies and emphasizing the importance of preventing and treating mental health problems during similar large-scale public health events such as COVID-19 (32). We observed disparities in the self-reported rates of anxiety and depressive symptoms between China and Korea, with the Chinese participants reporting a higher prevalence of anxiety symptoms and the Korean participants reporting a higher prevalence of depressive symptoms. This finding concurs with previous cross-cultural studies, suggesting that different cultural backgrounds might modulate individuals' appraisal and coping of psychological stress (5, 33–35). Some potential explanatory factors include cultural variations in social support, religious beliefs, values, emotional expression, etc. (34, 35); factual differences in COVID-19 prevention and control measures, medical resources, economic impact, etc. (36–38); and differences in the availability, acceptability, and quality of mental health services (18, 39–41). These factors might engender different magnitudes and types of psychological stress, thereby influencing the incidence of anxiety and depressive symptoms. Furthermore, we detected that different sociodemographic groups also had disparate self-reported rates of anxiety and depressive symptoms, implying that some subgroups might be more susceptible to COVID-19-related stressors. These subgroups might be deficient in adequate social support and resources, thus exacerbating their psychological stress and susceptibility to adverse mental health outcomes (42–45). Additionally, in this study, there were no significant differences in the prevalence of anxiety and depressive symptoms between participants with or without a history of COVID-19 infection, either in China or Korea. This might imply that COVID-19 infection did not exert a salient impact on the mental health of the Chinese and Korean people or that it was counteracted by other factors. For instance, the government offered free testing, isolation, treatment, and other services for infected people, as well as psychological counseling, health education, social assistance, and other support (36–38, 41). These measures might mitigate the economic burden and psychological pressure of infected people and foster their trust and belongingness toward the government and society.

Our results suggest that the Chinese population had a relatively higher psychological resilience than the Korean population, as indicated by their higher BRS score (3.34 ± 0.68 vs. 3.04 ± 0.71). This difference might be explained by the cultural factors that characterize the Chinese population, such as their stronger collectivistic orientation, higher sense of social responsibility, better adaptability, and greater self-efficacy (35, 46–48). Furthermore, the Chinese government adopted a series of effective measures during the COVID-19 pandemic, such as providing free testing, isolation, treatment, and other services, as well as offering psychological counseling, health education, and social assistance (18, 36–41). These measures might have fostered the trust and belongingness of the Chinese population toward the government and society, thus enhancing their psychological resilience.

A key finding of our study is that psychological resilience was significantly and inversely associated with anxiety and depressive symptoms, implying that higher psychological resilience might buffer the adverse effects of COVID-19-related stressors on mental health. This finding corroborates previous literature, suggesting that psychological resilience is a protective factor that can augment individuals' coping and recovery capacities in the face of adversity (20–22). We also observed that psychological resilience and mental health problems differed across various subgroups, indicating that some subgroups might be more susceptible to COVID-19-related stressors or more in need of bolstering their psychological resilience (20). For instance, among the Chinese participants, the subgroups with younger age, rural residence, lower education, unmarried status, living alone, renting housing, lower income, no young children, and chronic illness exhibited lower psychological resilience and a higher prevalence of mental distress. Among the Korean participants, people with unmarried status, renting housing, lower income, and chronic illness warranted special attention. These subgroups might encounter more challenges and uncertainties in economic, social, health, and other domains, especially during the pandemic, thus undermining their psychological resilience and mental health levels (42–45). Therefore, psychological resilience training and promotion strategies might be more essential and efficacious for these subgroups.

To our knowledge, this is the first cross-national study to compare psychological resilience and mental distress among Chinese and Korean populations amid the Omicron variant surge, generating valuable data and insights into mental health outcomes 3 years into the COVID-19 pandemic. This study revealed disparities in the prevalence and distribution of mental distress across China and Korea, which may inform the development of context-specific mental health policies and services. We also underscored the significance of fostering psychological resilience, which can offer guidance for devising effective resilience-enhancing and stress-reducing strategies to cope with the psychological challenges posed by the COVID-19 pandemic.

Despite these strengths, our study also has several limitations. First, our cross-sectional design precludes causal inference and warrants caution in interpreting the observed associations. Second, the online survey method may introduce response bias, as only those who had access to the internet could participate. However, we employed quota sampling based on the demographic characteristics of the two countries and used validated measurement tools. In addition, mental health symptoms in this study were based on self-reported results rather than clinical diagnosis. The self-reported results of the GAD-7 and PHQ-9 might not be enough for the investigation of anxiety and depression, and therefore, they should be interpreted with caution. Future studies are encouraged to adopt longitudinal or experimental designs to corroborate our findings and elucidate the potential mechanisms and interventions for psychological resilience and mental distress. Also, comparisons between different waves of COVID-19 or before and after the end of the pandemic may offer a more comprehensive understanding of the evolving mental health impacts.

## Conclusion

Amid the spread of the Omicron variant, Chinese and Korean populations reported a high prevalence of mental distress with variations in different characteristics. Chinese populations showed a relative advantage in psychological resilience, which was negatively associated with psychological distress, suggesting that higher resilience may buffer against the detrimental effects of COVID-19-related stressors on mental health. This study provides a cross-cultural perspective on psychological resilience and mental health in the context of the COVID-19 pandemic and has practical implications for developing tailored mental health policies and services, as well as designing effective resilience training and enhancement strategies, for populations with different cultural backgrounds.

## Data Availability

The datasets presented in this article are not readily available, because all data in the study are available from the corresponding author by request. The original data set is not available for the time being because of the need for other subsequent studies. Requests to access the datasets should be directed to jueliu@bjmu.edu.cn.
