# Supplementary material for: Mental health and psychological resilience amid the spread of the Omicron variant: a comparison between China and Korea
Source: Front Public Health. 2025 Jan 7;12:1451318. doi: 10.3389/fpubh.2024.1451318 (PMC11746007; doi:10.3389/fpubh.2024.1451318)
Supplement: Supplementary file 1 [file Table_1.docx]

| Table S1. Prevalence of anxiety and depressive symptoms among participants with COVID-19 history in China and Korea amid the spread of Omicron variant | | | | | | | | | | | | |
| --- | --- | --- | --- | --- | --- | --- | --- | --- | --- | --- | --- | --- |
| **Country** | **Characteristic** | **Anxiety symptom** | |  |  | **Depressive symptom** | | |  | **Any of the two** | | |
|  |  | **n (%)** | **95% CI** | **P value** |  | **n (%)** | **95% CI** | **P value** |  | **n (%)** | **95% CI** | **P value** |
| **China**  **(N=2690)** | **Total** | 671 (24.9) | 23.3-26.6 |  |  | 823 (30.6) | 28.9-32.4 |  |  | 963 (35.8) | 34.0-37.6 |  |
|  | **Sex** |  |  | 0.259 |  |  |  | 0.24 |  |  |  | 0.273 |
|  | Male | 254 (23.8) | 21.3-26.4 |  |  | 313 (29.3) | 26.6-32.1 |  |  | 369 (34.6) | 31.7-37.4 |  |
|  | Female | 417 (25.7) | 23.6-27.9 |  |  | 510 (31.4) | 29.2-33.7 |  |  | 594 (36.6) | 34.3-39.0 |  |
|  | **Age (years)** |  |  | <0.001* |  |  |  | <0.001* |  |  |  | <0.001* |
|  | <30 | 377 (30.5) | 28.0-33.1 |  |  | 451 (36.5) | 33.9-39.2 |  |  | 528 (42.8) | 40.0-45.5 |  |
|  | 30～39 | 250 (21.6) | 19.3-24.0 |  |  | 318 (27.4) | 24.9-30.0 |  |  | 370 (31.9) | 29.3-34.6 |  |
|  | 40～49 | 32 (14.7) | 10.5-19.8 |  |  | 39 (17.9) | 13.2-23.4 |  |  | 48 (22.0) | 16.9-27.9 |  |
|  | ≥50 | 12 (15.6) | 8.8-24.9 |  |  | 15 (19.5) | 11.8-29.4 |  |  | 17 (22.1) | 13.9-32.3 |  |
|  | **Location** |  |  | <0.001* |  |  |  | <0.001* |  |  |  | <0.001* |
|  | Urban | 370 (21.0) | 19.1-22.9 |  |  | 461 (26.1) | 24.1-26.1 |  |  | 547 (31.0) | 28.9-33.2 |  |
|  | Rural | 301 (32.5) | 29.5-35.6 |  |  | 362 (39.1) | 36.0-39.1 |  |  | 416 (44.9) | 41.7-48.1 |  |
|  | **Education†** |  |  | .008* |  |  |  | .015* |  |  |  | .001* |
|  | High school and below | 146 (30.1) | 26.1-34.3 |  |  | 173 (35.7) | 31.5-40.0 |  |  | 208 (42.9) | 38.5-47.3 |  |
|  | Bachelor degree | 472 (23.5) | 21.7-25.4 |  |  | 599 (29.8) | 27.8-31.8 |  |  | 686 (34.1) | 32.1-36.2 |  |
|  | Master degree | 53 (27.0) | 21.2-33.6 |  |  | 51 (26.0) | 20.3-32.5 |  |  | 69 (35.2) | 28.8-42.1 |  |
|  | **Marital status** |  |  | <0.001* |  |  |  | <0.001* |  |  |  | <0.001* |
|  | Unmarried | 218 (32.9) | 29.4-36.5 |  |  | 257 (38.8) | 35.1-42.5 |  |  | 298 (44.9) | 41.2-48.7 |  |
|  | Married | 445 (22.2) | 20.4-24.0 |  |  | 555 (27.7) | 25.7-29.7 |  |  | 652 (32.5) | 30.5-34.6 |  |
|  | Others | 8 (38.1) | 19.9-59.3 |  |  | 11 (52.4) | 31.9-72.3 |  |  | 13 (61.9) | 40.7-80.1 |  |
|  | **Household size** |  |  | <0.001* |  |  |  | .001* |  |  |  | <0.001* |
|  | Solitary | 71 (29.8) | 30.6-44.2 |  |  | 78 (36.4) | 34.0-47.9 |  |  | 90 (43.8) | 40.1-54.2 |  |
|  | 2 | 81 (22.5) | 24.6-35.4 |  |  | 99 (28.4) | 30.8-42.2 |  |  | 119 (33.0) | 37.9-49.7 |  |
|  | 3 | 267 (24.2) | 20.2-25.0 |  |  | 337 (29.7) | 25.9-31.0 |  |  | 391 (34.9) | 30.3-35.7 |  |
|  | ≥4 | 252 (23.1) | 21.7-26.9 |  |  | 309 (28.7) | 27.0-32.5 |  |  | 363 (33.5) | 32.0-37.8 |  |
|  | **Housing** |  |  | <0.001* |  |  |  | <0.001* |  |  |  | <0.001* |
|  | Purchased | 513 (23.1) | 21.4-24.9 |  |  | 637 (28.7) | 26.8-30.6 |  |  | 745 (33.5) | 31.6-35.5 |  |
|  | Rental | 158 (33.8) | 29.6-38.1 |  |  | 186 (39.7) | 35.4-44.2 |  |  | 218 (46.6) | 42.1-51.1 |  |
|  | **Income§** |  |  | <0.001* |  |  |  | <0.001* |  |  |  | <0.001* |
|  | Lower level | 62 (37.6) | 30.5-45.1 |  |  | 75 (45.5) | 38.0-53.1 |  |  | 84 (50.9) | 43.3-58.5 |  |
|  | Higher level | 609 (24.1) | 22.5-25.8 |  |  | 748 (29.6) | 27.9-31.4 |  |  | 879 (34.8) | 33.0-36.7 |  |
|  | **Young children**†† |  |  | <0.001* |  |  |  | <0.001* |  |  |  | <0.001* |
|  | No | 296 (30.6) | 27.8-33.6 |  |  | 345 (35.7) | 32.7-38.7 |  |  | 409 (42.3) | 39.2-45.4 |  |
|  | Yes | 375 (21.8) | 19.9-23.8 |  |  | 478 (27.7) | 25.7-29.9 |  |  | 554 (32.2) | 30.0-34.4 |  |
|  | **Chronic disease** |  |  | <0.001* |  |  |  | <0.001* |  |  |  | <0.001* |
|  | No | 421 (22.0) | 20.2-23.9 |  |  | 521 (27.3) | 25.3-29.3 |  |  | 619 (32.4) | 30.3-34.5 |  |
|  | Yes | 250 (32.1) | 28.9-35.4 |  |  | 302 (38.8) | 35.4-42.2 |  |  | 344 (44.2) | 40.7-47.7 |  |
|  | **Psychological resilience** |  |  | <0.001* |  |  |  | <0.001* |  |  |  | <0.001* |
|  | Low | 177 (64.6) | 58.8-70.1 |  |  | 191 (69.7) | 64.1-74.9 |  |  | 216 (78.8) | 73.7-83.4 |  |
|  | Moderate | 451 (27.4) | 25.3-29.6 |  |  | 591 (35.9) | 33.6-38.2 |  |  | 685 (41.6) | 39.3-44.0 |  |
|  | High | 43 (5.6) | 4.1-7.4 |  |  | 41 (5.3) | 3.9-7.1 |  |  | 62 (8.1) | 6.3-10.1 |  |
| **Korea (N=614)** | **Total** | 100 (16.3) | 13.5-19.4 |  |  | 204 (33.2) | 29.6-37.0 |  |  | 211 (34.4) | 30.7-38.2 |  |
|  | **Sex** |  |  | 0.12 |  |  |  | 0.772 |  |  |  | 0.613 |
|  | Male | 54 (18.8) | 14.6-23.6 |  |  | 94 (32.6) | 27.4-38.2 |  |  | 96 (33.3) | 28.1-38.9 |  |
|  | Female | 46 (14.1) | 10.7-18.2 |  |  | 110 (33.7) | 28.8-39.0 |  |  | 115 (35.3) | 30.2-40.6 |  |
|  | **Age (years)** |  |  | .009* |  |  |  | 0.198 |  |  |  | 0.172 |
|  | <30 | 27 (21.1) | 14.7-28.8 |  |  | 47 (36.7) | 28.7-45.3 |  |  | 50 (39.1) | 30.9-47.7 |  |
|  | 30～39 | 21 (20.4) | 13.5-28.9 |  |  | 35 (34.0) | 25.4-43.5 |  |  | 35 (34.0) | 25.4-43.5 |  |
|  | 40～49 | 24 (20.7) | 14.1-28.7 |  |  | 45 (38.8) | 30.3-47.8 |  |  | 46 (39.7) | 31.1-48.7 |  |
|  | ≥50 | 28 (10.5) | 7.2-14.6 |  |  | 77 (28.8) | 23.7-34.5 |  |  | 80 (30.0) | 24.7-35.7 |  |
|  | **Location** |  |  | 0.559 |  |  |  | 0.982 |  |  |  | 0.967 |
|  | Urban | 85 (15.9) | 13.0-19.2 |  |  | 177 (33.2) | 29.3-37.3 |  |  | 183 (34.3) | 30.4-38.4 |  |
|  | Rural | 15 (18.5) | 11.2-28.0 |  |  | 27 (33.3) | 23.8-44.0 |  |  | 28 (34.6) | 24.9-45.3 |  |
|  | **Education†** |  |  | 0.098 |  |  |  | 0.34 |  |  |  | 0.276 |
|  | High school and below | 52 (15.6) | 12.0-19.8 |  |  | 113 (33.9) | 29.0-39.1 |  |  | 118 (35.4) | 30.4-40.7 |  |
|  | Bachelor degree | 47 (18.7) | 14.2-23.8 |  |  | 85 (33.7) | 28.1-39.7 |  |  | 87 (34.5) | 28.9-40.5 |  |
|  | Master degree | 1 (3.4) | 0.4-15.0 |  |  | 6 (20.7) | 9.1-37.8 |  |  | 6 (20.7) | 9.1-37.8 |  |
|  | **Marital status** |  |  | 0.063 |  |  |  | .034* |  |  |  | 0.037* |
|  | Unmarried | 43 (21.3) | 16.1-27.3 |  |  | 77 (38.1) | 31.6-44.9 |  |  | 80 (39.6) | 33.0-46.5 |  |
|  | Married | 50 (13.9) | 10.6-17.7 |  |  | 105 (29.2) | 24.7-34.0 |  |  | 109 (30.3) | 25.7-35.2 |  |
|  | Others | 7 (13.5) | 6.2-24.6 |  |  | 22 (42.3) | 29.6-55.8 |  |  | 22 (42.3) | 29.6-55.8 |  |
|  | **Household size** |  |  | 0.106 |  |  |  | .020* |  |  |  | .027* |
|  | Solitary | 17 (20.5) | 12.9-30.1 |  |  | 36 (43.4) | 33.1-54.1 |  |  | 36 (43.4) | 33.1-54.1 |  |
|  | 2 | 32 (20.8) | 15.0-27.7 |  |  | 56 (36.4) | 29.1-44.2 |  |  | 58 (37.7) | 30.3-45.5 |  |
|  | 3 | 27 (15.2) | 10.5-21.0 |  |  | 61 (34.3) | 27.6-41.5 |  |  | 64 (36.0) | 29.2-43.2 |  |
|  | ≥4 | 24 (12.1) | 8.1-17.1 |  |  | 51 (25.6) | 19.9-32.0 |  |  | 53 (26.6) | 20.9-33.1 |  |
|  | **Housing** |  |  | 0.007* |  |  |  | 0.007* |  |  |  | 0.010* |
|  | Purchased | 51 (13.2) | 10.1-16.9 |  |  | 113 (29.3) | 24.9-34.0 |  |  | 118 (30.6) | 26.1-35.3 |  |
|  | Rental | 49 (21.5) | 16.5-27.2 |  |  | 91 (39.9) | 33.7-46.4 |  |  | 93 (40.8) | 34.6-47.3 |  |
|  | **Income§** |  |  | 0.016* |  |  |  | .003* |  |  |  | .004* |
|  | Lower level | 42 (21.5) | 16.2-27.7 |  |  | 81 (41.5) | 34.8-48.5 |  |  | 83 (42.6) | 35.8-49.6 |  |
|  | Higher level | 58 (13.8) | 10.8-17.4 |  |  | 123 (29.4) | 25.1-33.8 |  |  | 128 (30.5) | 26.3-35.1 |  |
|  | **Young children**†† |  |  | 0.649 |  |  |  | 0.645 |  |  |  | 0.842 |
|  | No | 63 (15.8) | 12.5-19.6 |  |  | 130 (32.6) | 28.1-37.3 |  |  | 136 (34.1) | 29.6-38.8 |  |
|  | Yes | 37 (17.2) | 12.6-22.7 |  |  | 74 (34.4) | 28.3-40.9 |  |  | 75 (34.9) | 28.7-41.4 |  |
|  | **Chronic disease** |  |  | 0.022* |  |  |  | 0.081 |  |  |  | 0.208 |
|  | No | 51 (13.6) | 10.4-17.3 |  |  | 115 (30.6) | 26.1-35.4 |  |  | 122 (32.4) | 27.9-37.3 |  |
|  | Yes | 49 (20.6) | 15.8-26.1 |  |  | 89 (37.4) | 31.4-43.7 |  |  | 89 (37.4) | 31.4-43.7 |  |
|  | **Psychological resilience** |  |  | <0.001* |  |  |  | <0.001* |  |  |  | <0.001* |
|  | Low | 41 (38.0) | 29.2-47.3 |  |  | 64 (59.3) | 49.8-68.2 |  |  | 66 (61.1) | 51.7-69.9 |  |
|  | Moderate | 54 (13.4) | 10.4-17.0 |  |  | 130 (32.3) | 27.9-37.0 |  |  | 134 (33.3) | 28.9-38.0 |  |
|  | High | 5 (4.8) | 1.9-10.2 |  |  | 10 (9.6) | 5.1-16.4 |  |  | 11 (10.6) | 5.8-17.6 |  |
| * A P value less than 0.05 is considered to be statistically significant.  **†** Junior college in China was assigned to “high school and below” in our analyses.  § We divided the income brackets based on the official average monthly income for China and Korea in 2022. Combining our questionnaire setup, we set income below 3000 RMB (China) and 3 million won (Korea) as the “Lower level”.  †† Young children were defined as children attending high school or younger. | | | | | | | | | | | | |

| Table S2. Prevalence of anxiety and depressive symptoms among participants without COVID-19 history in China and Korea amid the spread of Omicron variant | | | | | | | | | | | | |
| --- | --- | --- | --- | --- | --- | --- | --- | --- | --- | --- | --- | --- |
| **Country** | **Characteristics** | **Anxiety symptom** | | |  | **Depressive symptom** | | |  | **Any of the two** | | |
|  |  | **n (%)** | **95% CI** | **P value** |  | **n (%)** | **95% CI** | **P value** |  | **n (%)** | **95% CI** | **P value** |
| **China**  **(N=310)** | **Total** | 64 (20.6) | 16.4-25.4 |  |  | 91 (29.4) | 24.5-34.6 |  |  | 102 (32.9) | 27.9-38.3 |  |
|  | **Sex** |  |  | 0.037* |  |  |  | 0.308 |  |  |  | 0.107 |
|  | Male | 25 (15.9) | 10.8-22.2 |  |  | 42 (26.8) | 20.3-34.1 |  |  | 45 (28.7) | 22.0-36.1 |  |
|  | Female | 39 (25.5) | 19.1-32.8 |  |  | 49 (32.0) | 25.0-39.7 |  |  | 57 (37.3) | 29.9-45.1 |  |
|  | **Age (years)** |  |  | 0.097 |  |  |  | 0.215 |  |  |  | 0.136 |
|  | <30 | 39 (26.0) | 19.5-33.4 |  |  | 52 (34.7) | 27.4-42.5 |  |  | 59 (39.3) | 31.8-47.3 |  |
|  | 30～39 | 16 (14.4) | 8.8-21.8 |  |  | 28 (25.2) | 17.9-33.9 |  |  | 29 (26.1) | 18.6-34.8 |  |
|  | 40～49 | 7 (22.6) | 10.7-39.3 |  |  | 6 (19.4) | 8.5-35.6 |  |  | 9 (29.0) | 15.4-46.3 |  |
|  | ≥50 | 2 (11.1) | 2.4-31.1 |  |  | 5 (27.8) | 11.5-50.6 |  |  | 5 (27.8) | 11.5-50.6 |  |
|  | **Location** |  |  | 0.048* |  |  |  | 0.049* |  |  |  | 0.061 |
|  | Urban | 33 (17.1) | 12.3-22.9 |  |  | 49 (25.4) | 19.6-25.4 |  |  | 56 (29.0) | 23.0-35.7 |  |
|  | Rural | 31 (26.5) | 19.1-35.0 |  |  | 42 (35.9) | 27.6-35.9 |  |  | 46 (39.3) | 30.8-48.3 |  |
|  | **Education†** |  |  | 0.642 |  |  |  | 0.007* |  |  |  | 0.006* |
|  | High school and below | 16 (23.9) | 14.9-35.0 |  |  | 30 (44.8) | 33.3-56.7 |  |  | 33 (49.3) | 37.5-61.0 |  |
|  | Bachelor degree | 44 (20.3) | 15.3-26.0 |  |  | 55 (25.3) | 19.9-31.4 |  |  | 62 (28.6) | 22.9-34.8 |  |
|  | Master degree | 4 (15.4) | 5.4-32.5 |  |  | 6 (23.1) | 10.3-41.5 |  |  | 7 (26.9) | 12.9-45.7 |  |
|  | **Marital status**¶ |  |  | 0.009* |  |  |  | 0.020* |  |  |  | 0.005* |
|  | Unmarried | 33 (29.7) | 21.8-38.7 |  |  | 42 (37.8) | 29.2-47.1 |  |  | 48 (43.2) | 34.3-52.5 |  |
|  | Married | 31 (15.7) | 11.2-21.3 |  |  | 48 (24.4) | 18.8-30.7 |  |  | 53 (26.9) | 21.1-33.4 |  |
|  | Others | 0 (0.0) | / |  |  | 1 (50.0) | 6.1-93.9 |  |  | 1 (50.0) | 6.1-93.9 |  |
|  | **Household size** |  |  | 0.91 |  |  |  | 0.475 |  |  |  | 0.332 |
|  | Solitary | 9 (18.5) | 13.2-40.7 |  |  | 14 (25.9) | 24.3-55.2 |  |  | 15 (25.9) | 26.7-57.9 |  |
|  | 2 | 5 (20.6) | 7.4-35.9 |  |  | 7 (30.5) | 12.4-44.3 |  |  | 7 (35.9) | 12.4-44.3 |  |
|  | 3 | 27 (19.8) | 14.4-28.1 |  |  | 40 (25.9) | 23.1-38.8 |  |  | 47 (28.4) | 28.0-44.3 |  |
|  | ≥4 | 23 (18.1) | 13.4-27.8 |  |  | 30 (26.3) | 18.6-34.4 |  |  | 33 (29.2) | 20.8-37.1 |  |
|  | **Housing** |  |  | 0.035* |  |  |  | 0.026* |  |  |  | 0.009* |
|  | Purchased | 44 (18.1) | 13.7-23.3 |  |  | 64 (26.3) | 21.1-32.1 |  |  | 71 (29.2) | 23.8-35.2 |  |
|  | Rental | 20 (29.9) | 19.9-41.5 |  |  | 27 (40.3) | 29.2-52.2 |  |  | 31 (46.3) | 34.7-58.2 |  |
|  | **Income§** |  |  | 0.039* |  |  |  | 0.038* |  |  |  | 0.015* |
|  | Lower level | 10 (35.7) | 20.1-54.2 |  |  | 13 (46.4) | 29.1-64.5 |  |  | 15 (53.6) | 35.5-70.9 |  |
|  | Higher level | 54 (19.1) | 14.9-24.0 |  |  | 78 (27.7) | 22.7-33.1 |  |  | 87 (30.9) | 25.7-36.4 |  |
|  | **Young children**†† |  |  | 0.018* |  |  |  | 0.059 |  |  |  | 0.044* |
|  | No | 39 (26.4) | 19.8-33.9 |  |  | 51 (34.5) | 27.2-42.4 |  |  | 57 (38.5) | 31.0-46.5 |  |
|  | Yes | 25 (15.4) | 10.5-21.6 |  |  | 40 (24.7) | 18.5-31.7 |  |  | 45 (27.8) | 21.3-35.0 |  |
|  | **Chronic disease** |  |  | 0.62 |  |  |  | 0.199 |  |  |  | 0.232 |
|  | No | 50 (20.1) | 15.5-25.4 |  |  | 69 (27.7) | 22.4-33.5 |  |  | 78 (31.3) | 25.8-37.3 |  |
|  | Yes | 14 (23.0) | 13.8-34.6 |  |  | 22 (36.1) | 24.9-48.5 |  |  | 24 (39.3) | 27.8-51.9 |  |
|  | **Psychological resilience** |  |  | <0.001* |  |  |  | <0.001* |  |  |  | <0.001* |
|  | Low | 20 (58.8) | 42.1-74.1 |  |  | 24 (70.6) | 54.1-83.8 |  |  | 24 (70.6) | 54.1-83.8 |  |
|  | Moderate | 38 (21.7) | 16.1-28.3 |  |  | 60 (34.3) | 27.6-41.5 |  |  | 67 (38.3) | 31.3-45.6 |  |
|  | High | 6 (5.9) | 2.5-11.8 |  |  | 7 (6.9) | 3.2-13.1 |  |  | 11 (10.9) | 5.9-18.1 |  |
| **Korea (N=386)** | **Total** | 72 (18.7) | 15.0-22.8 |  |  | 140 (36.3) | 31.6-41.2 |  |  | 146 (37.8) | 33.1-42.7 |  |
|  | **Sex** |  |  | 0.716 |  |  |  | 0.987 |  |  |  | 0.785 |
|  | Male | 40 (19.3) | 14.4-25.1 |  |  | 75 (36.2) | 29.9-42.9 |  |  | 77 (37.2) | 30.8-43.9 |  |
|  | Female | 32 (17.9) | 12.8-24.0 |  |  | 65 (36.3) | 29.5-43.5 |  |  | 69 (38.5) | 31.6-45.8 |  |
|  | **Age (years)** |  |  | 0.004* |  |  |  | 0.281 |  |  |  | 0.135 |
|  | <30 | 9 (23.7) | 12.4-38.8 |  |  | 15 (39.5) | 25.2-55.3 |  |  | 15 (39.5) | 25.2-55.3 |  |
|  | 30～39 | 15 (32.6) | 20.4-46.9 |  |  | 22 (47.8) | 33.9-62.0 |  |  | 23 (50.0) | 35.9-64.1 |  |
|  | 40～49 | 17 (25.4) | 16.1-36.7 |  |  | 25 (37.3) | 26.5-49.2 |  |  | 29 (43.3) | 31.9-55.2 |  |
|  | ≥50 | 31 (13.2) | 9.3-18.0 |  |  | 78 (33.2) | 27.4-39.4 |  |  | 79 (33.6) | 27.8-39.8 |  |
|  | **Location** |  |  | 0.351 |  |  |  | 0.586 |  |  |  | 0.409 |
|  | Urban | 57 (17.8) | 13.9-22.3 |  |  | 118 (36.9) | 31.7-42.3 |  |  | 124 (38.8) | 33.5-44.2 |  |
|  | Rural | 15 (22.7) | 13.9-33.9 |  |  | 22 (33.3) | 22.9-45.2 |  |  | 22 (33.3) | 22.9-45.2 |  |
|  | **Education†** |  |  | 0.432 |  |  |  | 0.32 |  |  |  | 0.467 |
|  | High school and below | 48 (20.0) | 15.3-25.4 |  |  | 92 (38.3) | 32.4-44.6 |  |  | 96 (40.0) | 34.0-46.3 |  |
|  | Bachelor degree | 21 (15.6) | 10.2-22.4 |  |  | 46 (34.1) | 26.5-42.3 |  |  | 47 (34.8) | 27.2-43.1 |  |
|  | Master degree | 3 (27.3) | 8.3-56.5 |  |  | 2 (18.2) | 4.0-46.7 |  |  | 3 (27.3) | 8.3-56.5 |  |
|  | **Marital status** |  |  | 0.051 |  |  |  | 0.146 |  |  |  | 0.032* |
|  | Unmarried | 32 (25.6) | 18.6-33.7 |  |  | 54 (43.2) | 34.8-52.0 |  |  | 59 (47.2) | 38.6-55.9 |  |
|  | Married | 31 (15.0) | 10.6-20.3 |  |  | 68 (32.9) | 26.7-39.5 |  |  | 69 (33.3) | 27.2-40.0 |  |
|  | Others | 9 (16.7) | 8.6-28.2 |  |  | 18 (33.3) | 21.9-46.5 |  |  | 18 (33.3) | 21.9-46.5 |  |
|  | **Household size** |  |  | 0.236 |  |  |  | 0.856 |  |  |  | 0.635 |
|  | Solitary | 20 (26.0) | 17.2-36.5 |  |  | 31 (40.3) | 29.8-51.4 |  |  | 34 (44.2) | 33.4-55.3 |  |
|  | 2 | 20 (15.0) | 9.7-21.8 |  |  | 47 (35.3) | 27.6-43.7 |  |  | 49 (36.8) | 29.0-45.3 |  |
|  | 3 | 17 (20.0) | 12.6-29.4 |  |  | 31 (36.5) | 26.8-47.0 |  |  | 31 (36.5) | 26.8-47.0 |  |
|  | ≥4 | 15 (16.5) | 10.0-25.1 |  |  | 31 (34.1) | 24.9-44.2 |  |  | 32 (35.2) | 25.9-45.3 |  |
|  | **Housing** |  |  | 0.708 |  |  |  | 0.002* |  |  |  | 0.006* |
|  | Purchased | 48 (19.2) | 14.7-24.4 |  |  | 77 (30.8) | 25.3-36.7 |  |  | 82 (32.8) | 27.2-38.8 |  |
|  | Rental | 24 (17.6) | 12.0-24.7 |  |  | 63 (46.3) | 38.1-54.7 |  |  | 64 (47.1) | 38.8-55.4 |  |
|  | **Income§** |  |  | 0.018* |  |  |  | 0.042* |  |  |  | 0.033* |
|  | Lower level | 38 (24.4) | 18.1-31.5 |  |  | 66 (42.3) | 34.8-50.1 |  |  | 69 (44.2) | 36.6-52.1 |  |
|  | Higher level | 34 (14.8) | 10.6-19.8 |  |  | 74 (32.2) | 26.4-38.4 |  |  | 77 (33.5) | 27.6-39.8 |  |
|  | **Young children**†† |  |  | 0.966 |  |  |  | 0.455 |  |  |  | 0.257 |
|  | No | 52 (18.7) | 14.5-23.6 |  |  | 104 (37.4) | 31.9-43.2 |  |  | 110 (39.6) | 34.0-45.4 |  |
|  | Yes | 72 (18.7) | 15.0-22.8 |  |  | 36 (33.3) | 25.0-42.6 |  |  | 36 (33.3) | 25.0-42.6 |  |
|  | **Chronic disease** |  |  | 0.405 |  |  |  | 0.031* |  |  |  | 0.076 |
|  | No | 36 (17.1) | 12.5-22.7 |  |  | 66 (31.4) | 25.4-37.9 |  |  | 71 (33.8) | 27.7-40.4 |  |
|  | Yes | 36 (20.5) | 15.0-26.9 |  |  | 74 (42.0) | 34.9-49.4 |  |  | 75 (42.6) | 35.5-50.0 |  |
|  | **Psychological resilience** |  |  | <0.001* |  |  |  | <0.001* |  |  |  | <0.001* |
|  | Low | 30 (35.7) | 26.1-46.3 |  |  | 55 (65.5) | 54.9-75.0 |  |  | 56 (66.7) | 56.2-76.1 |  |
|  | Moderate | 40 (16.9) | 12.5-22.0 |  |  | 82 (34.6) | 28.8-40.8 |  |  | 86 (36.3) | 30.4-42.5 |  |
|  | High | 2 (3.1) | 0.6-9.5 |  |  | 3 (4.6) | 1.3-11.8 |  |  | 4 (6.2) | 2.1-14.0 |  |
| * A P value less than 0.05 is considered to be statistically significant.  **†** Junior college in China was assigned to “high school and below” in our analyses.  § We divided the income brackets based on the official average monthly income for China and Korea in 2022. Combining our questionnaire setup, we set income below 3000 RMB (China) and 3 million won (Korea) as the “Lower level”.  ¶ Fisher’s exact test.  †† Young children were defined as children attending high school or younger. | | | | | | | | | | | | |

| Table S3. Association between psychological resilience and mental distress among participants with or without COVID-19 history in China and Korea amid the spread of Omicron variant | | | | | | | | |  |
| --- | --- | --- | --- | --- | --- | --- | --- | --- | --- |
| **Participants** | **Models** | **China**** | | |  | **Korea**** | | |  |
|  |  | **Low resilience** |  | **Moderate resilience** |  | **Low resilience** |  | **Moderate resilience** |  |
|  |  | **RR (95% CI)** |  | **RR (95% CI)** |  | **RR (95% CI)** |  | **RR (95% CI)** |  |
| Participants with COVID-19 history | **Model A** |  |  |  |  |  |  |  |  |
|  | Anxiety symptom | 25.05 (9.47, 66.26) * |  | 8.58 (3.24, 22.76) * |  | / |  | / |  |
|  | Depressive symptom | 13.42 (7.07, 25.47) * |  | 4.72 (2.48, 9.00) * |  | 10.65 (1.57, 72.42) * |  | 5.22 (0.77, 35.65) |  |
|  | Any of the two | 13.71 (7.48, 25.13) * |  | 5.15 (2.80, 9.47) * |  | 11.04 (1.62, 75.05) * |  | 5.39 (0.79, 36.77) |  |
|  | **Model B** |  |  |  |  |  |  |  |  |
|  | Anxiety symptom | 21.87 (8.26, 57.95) * |  | 7.94 (3.00, 21.05) * |  | / |  | / |  |
|  | Depressive symptom | 11.85 (6.24, 22.51) * |  | 4.40 (2.31, 8.36) * |  | 10.18 (1.42, 72.79) * |  | 5.42 (0.75, 39.08) |  |
|  | Any of the two | 12.21 (6.66, 22.39) * |  | 4.82 (2.63, 8.85) * |  | 10.54 (1.48, 75.01) * |  | 5.55 (0.77, 39.86) |  |
|  | **Model C** |  |  |  |  |  |  |  |  |
|  | Anxiety symptom | 20.19 (7.62, 53.49) * |  | 7.57 (2.86, 20.04) * |  | / |  | / |  |
|  | Depressive symptom | 11.02 (5.80, 20.94) * |  | 4.21 (2.21, 8.00) * |  | 9.62 (1.34, 69.12) * |  | 5.13 (0.71, 37.23) |  |
|  | Any of the two | 11.46 (6.25, 21.02) * |  | 4.64 (2.53, 8.52) * |  | 10.09 (1.41, 72.12) * |  | 5.33 (0.74, 38.43) |  |
|  | **Model D** |  |  |  |  |  |  |  |  |
|  | Anxiety symptom | 20.19 (7.62, 53.49) * |  | 7.57 (2.86, 20.04) * |  | / |  | / |  |
|  | Depressive symptom | 11.02 (5.80, 20.94) * |  | 4.21 (2.21, 8.00) * |  | 9.62 (1.34, 69.12) * |  | 5.13 (0.71, 37.23) |  |
|  | Any of the two | 11.46 (6.25, 21.02) * |  | 4.64 (2.53, 8.52) * |  | 10.09 (1.41, 72.12) * |  | 5.33 (0.74, 38.43) |  |
| Participants without COVID-19 history | **Model A** |  |  |  |  |  |  |  |  |
|  | Anxiety symptom | 11.74 (1.68, 81.95) * |  | 4.27 (0.60, 30.16) |  | 4.55 (0.68, 30.67) |  | 1.48 (0.21, 10.27) |  |
|  | Depressive symptom | 17.61 (2.55, 121.74) * |  | 5.74 (0.82, 40.20) |  | 4.15 (1.14, 15.19) * |  | 1.66 (0.45, 6.20) |  |
|  | Any of the two | 9.15 (2.38, 35.19) * |  | 3.46 (0.89, 13.48) |  | 4.38 (1.20, 16.00) * |  | 1.70 (0.46, 6.33) |  |
|  | **Model B** |  |  |  |  |  |  |  |  |
|  | Anxiety symptom | 10.01 (1.40, 71.34) * |  | 3.88 (0.55, 27.61) |  | 4.97 (0.77, 32.19) |  | 1.59 (0.24, 10.43) |  |
|  | Depressive symptom | 15.10 (2.20, 103.76) * |  | 5.43 (0.80, 36.72) |  | 4.00 (1.13, 14.07) * |  | 1.58 (0.44, 5.64) |  |
|  | Any of the two | 7.49 (2.01, 27.97) * |  | 3.18 (0.86, 11.78) |  | 4.29 (1.20, 15.32) * |  | 1.64 (0.45, 5.93) |  |
|  | **Model C** |  |  |  |  |  |  |  |  |
|  | Anxiety symptom | 9.81 (1.42, 67.89) * |  | 3.78 (0.54, 26.31) |  | 4.26 (0.65, 27.93) * |  | 1.39 (0.21, 9.26) |  |
|  | Depressive symptom | 14.59 (2.13, 99.92) * |  | 5.27 (0.78, 35.57) |  | 3.57 (1.05, 12.19) * |  | 1.44 (0.42, 4.97) |  |
|  | Any of the two | 7.30 (2.01, 26.48) * |  | 3.10 (0.86, 11.18) |  | 3.91 (1.12, 13.65) * |  | 1.51 (0.43, 5.35) |  |
|  | **Model D** |  |  |  |  |  |  |  |  |
|  | Anxiety symptom | 9.81 (1.42, 67.89) * |  | 3.78 (0.54, 26.31) |  | 4.26 (0.65, 27.93) |  | 1.39 (0.21, 9.26) |  |
|  | Depressive symptom | 14.59 (2.13, 99.92) * |  | 5.27 (0.78, 35.57) |  | 3.57 (1.05, 12.19) * |  | 1.44 (0.42, 4.97) |  |
|  | Any of the two | 7.30 (2.01, 26.48) * |  | 3.10 (0.86, 11.18) |  | 3.91 (1.12, 13.65) * |  | 1.51 (0.43, 5.35) |  |
| * A P value less than 0.05 is considered to be statistically significant.  ** The high resilience group were set as the reference group in all models. Model A is a univariable modified Poisson regression model.  Model B is adjusted by sociodemographic characteristics (age; gender; location; education; marital status; household size; housing; income; children). Model C is adjusted by sociodemographic characteristics and chronic disease history. Model D is adjusted by sociodemographic characteristics, chronic disease history, and COVID-19 history. | | | | | | | | | |

| Table S4. Subgroup analysis of the association between psychological resilience and mental distress in China | | | | | | | | | |  | | |
| --- | --- | --- | --- | --- | --- | --- | --- | --- | --- | --- | --- | --- |
| **Characteristic** | **Anxiety symptom**** | | |  | **Depressive symptom**** | | |  | **Any of the two**** | | | |
|  | Low  resilience | Moderate resilience | P value |  | Low  resilience | Moderate resilience | P value |  | Low  resilience | Moderate  resilience | P value |  |
| **Sex** |  |  | 0.913 |  |  |  | 0.795 |  |  |  | 0.898 |  |
| Male | 15.40 (4.99, 47.52) | 5.93 (1.92, 18.27) |  |  | 12.13 (5.10, 28.83) | 4.40 (1.85, 10.47) |  |  | 9.76 (4.73, 20.12) | 3.89 (1.88, 8.03) |  |  |
| Female | 22.55 (5.66, 89.83) | 8.36 (2.10, 33.24) |  |  | 10.86 (4.58, 25.74) | 4.21 (1.78, 9.98) |  |  | 12.55 (5.31, 29.71) | 5.11 (2.16, 12.09) |  |  |
| **Age (years)** |  |  | 0.107 |  |  |  | 0.183 |  |  |  | 0.041* |  |
| <30 | 11.75 (3.83, 36.00) | 4.73 (1.54, 14.51) |  |  | 8.44 (3.59, 19.84) | 3.46 (1.47, 8.15) |  |  | 8.16 (3.77, 17.66) | 3.55 (1.64, 7.71) |  |  |
| 30～39 | 43.31 (6.13, 306.20) | 16.05 (2.27, 113.37) |  |  | 11.93 (3.77, 17.66) | 4.20 (1.64, 7.71) |  |  | 13.14 (5.53, 31.18) | 5.04 (2.12, 11.98) |  |  |
| 40～49 | 14.67 (2.60, 82.85) | 4.46 (0.74, 26.94) |  |  | / | / |  |  | 17.12 (4.05, 72.50) | 6.52 (1.50, 28.35) |  |  |
| ≥50 | / | / |  |  | / | / |  |  | / | / |  |  |
| **Location** |  |  | 0.001* |  |  |  | <0.001* |  |  |  | <0.001* |  |
| Urban | 38.38 (9.64, 152.90) | 12.10 (3.03, 48.31) |  |  | 14.35 (6.91, 29.83) | 4.33 (2.08, 9.02) |  |  | 13.98 (7.08, 27.62) | 4.77 (2.41, 9.44) |  |  |
| Rural | 7.04 (2.28, 21.73) | 3.21 (1.04, 9.94) |  |  | 8.44 (2.73, 26.16) | 4.14 (1.34, 12.81) |  |  | 7.07 (2.72, 18.36) | 3.50 (1.35, 9.11) |  |  |
| **Education†** |  |  | 0.509 |  |  |  | 0.110 |  |  |  | 0.116 |  |
| High school and below | 15.89 (2.32, 109.00) | 6.86 (1.00, 47.20) |  |  | 10.02 (2.56, 39.21) | 4.56 (1.17, 17.79) |  |  | 7.58 (2.59, 22.16) | 3.66 (1.25, 10.68) |  |  |
| Bachelor degree | 16.23 (6.13, 43.02) | 5.98 (2.26, 15.83) |  |  | 10.70 (5.41, 21.16) | 3.92 (1.98, 7.76) |  |  | 10.65 (5.62, 20.18) | 4.15 (2.18, 7.87) |  |  |
| Master degree | / | / |  |  | / | / |  |  | / | / |  |  |
| **Marital status** |  |  | 0.474 |  |  |  | 0.357 |  |  |  | 0.390 |  |
| Unmarried | 13.11 (1.86, 92.19) | 5.50 (0.78, 38.73) |  |  | 7.15 (1.87, 27.32) | 3.09 (0.80, 11.84) |  |  | 8.49 (2.21, 32.60) | 3.85 (1.00, 14.84) |  |  |
| Married | 20.07 (7.53, 53.46) | 7.29 (2.74, 19.41) |  |  | 13.08 (6.59, 25.96) | 4.66 (2.34, 9.26) |  |  | 11.70 (6.36, 21.52) | 4.51 (2.45, 8.31) |  |  |
| Others | / | / |  |  | 6.39 (0.58, 70.67) | / |  |  | 3.22 (0.48, 21.75) | / |  |  |
| **Household size** |  |  | 0.832 |  |  |  | 0.201 |  |  |  | 0.401 |  |
| Solitary | 4.92 (0.58, 41.44) | 1.77 (0.21, 14.59) |  |  | 2.20 (0.59, 8.25) | 0.98 (0.26, 3.67) |  |  | 2.49 (0.68, 9.07) | 1.13 (0.31, 4.10) |  |  |
| 2 | / | / |  |  | 9.97 (1.41, 70.61) | 4.19 (0.59, 29.84) |  |  | 11.23 (1.60, 78.86) | 5.14 (0.73, 36.32) |  |  |
| 3 | 19.83 (4.97, 79.19) | 7.24 (1.81, 28.88) |  |  | 17.98 (5.87, 55.06) | 6.20 (2.02, 19.05) |  |  | 14.90 (5.68, 39.11) | 5.76 (2.19, 15.14) |  |  |
| ≥4 | 18.81 (4.71, 75.09) | 7.23 (1.82, 28.81) |  |  | 11.44 (4.33, 30.20) | 4.51 (1.71, 11.89) |  |  | 10.62 (4.50, 25.06) | 4.29 (1.82, 10.13) |  |  |
| **Housing** |  |  | 0.010* |  |  |  | 0.012* |  |  |  | 0.001* |  |
| Purchased | 28.39 (9.20, 87.59) | 10.02 (3.24, 30.94) |  |  | 13.58 (6.86, 26.87) | 4.78 (2.41, 9.48) |  |  | 13.83 (7.29, 26.25) | 5.21 (2.74, 9.91) |  |  |
| Rental | 4.21 (1.17, 15.11) | 1.98 (0.55, 7.11) |  |  | 4.85 (1.28, 18.36) | 2.34 (0.62, 8.91) |  |  | 3.64 (1.38, 9.59) | 1.87 (0.71, 4.95) |  |  |
| **Income§** |  |  | 0.100 |  |  |  | 0.465 |  |  |  | 0.587 |  |
| Lower level | / | / |  |  | / | / |  |  | / | / |  |  |
| Higher level | 18.29 (7.65, 43.75) | 6.69 (2.80, 16.00) |  |  | 11.43 (6.21, 21.04) | 4.28 (2.33, 7.88) |  |  | 10.85 (6.24, 18.87) | 4.37 (2.51, 7.61) |  |  |
| **Young children††** |  |  | 0.696 |  |  |  | 0.328 |  |  |  | 0.273 |  |
| No | 23.46 (3.33, 165.07) | 9.34 (1.33, 65.49) |  |  | 8.57 (2.85, 25.78) | 3.56 (1.18, 10.72) |  |  | 10.36 (3.44, 31.18) | 4.55 (1.51, 13.73) |  |  |
| Yes | 16.70 (6.30, 44.32) | 6.17 (2.32, 16.36) |  |  | 12.73 (6.13, 26.41) | 4.57 (2.20, 9.50) |  |  | 11.02 (5.82, 20.87) | 4.27 (2.25, 8.10) |  |  |
| **Chronic disease** |  |  | 0.165 |  |  |  | 0.054 |  |  |  | 0.018* |  |
| No | 22.42 (7.25, 69.36) | 7.91 (2.56, 24.47) |  |  | 10.87 (5.49, 21.50) | 3.79 (1.91, 7.50) |  |  | 11.15 (5.88, 21.14) | 4.16 (2.19, 7.90) |  |  |
| Yes | 12.19 (3.13, 47.43) | 5.21 (1.33, 20.36) |  |  | 14.51 (3.70, 56.90) | 6.37 (1.62, 25.07) |  |  | 10.68 (3.58, 31.85) | 5.08 (1.70, 15.20) |  |  |
| **COVID-19 history** |  |  | 0.773 |  |  |  | 0.928 |  |  |  | 0.860 |  |
| No | 9.81 (1.42, 67.89) | 3.78 (0.54, 26.31) |  |  | 14.59 (2.13, 99.92) | 5.27 (0.78, 35.57) |  |  | 7.30 (2.01, 26.48) | 3.10 (0.86, 11.18) |  |  |
| Yes | 20.19 (7.62, 53.49) | 7.57 (2.86, 20.04) |  |  | 11.02 (5.80, 20.94) | 4.21 (2.21, 8.00) |  |  | 11.46 (6.25, 21.02) | 4.64 (2.53, 8.52) |  |  |
| * A P value for interaction less than 0.05 is considered to be statistically significant.  **†** Junior college in China was assigned to “high school and below” in our analyses. § We divided the income brackets based on the official average monthly income for China and Korea in 2022. Combining our questionnaire setup, we set income below 3000 RMB (China) and 3 million won (Korea) as the “Lower level”.  †† Young children were defined as children attending high school or younger.  ** The high resilience group were set as the reference group in all models. | | | | | | | | | | | | |

| Table S5. Subgroup analysis of the association between psychological resilience and mental distress in Korea | | | | | | | | | |  | | |
| --- | --- | --- | --- | --- | --- | --- | --- | --- | --- | --- | --- | --- |
| **Characteristic** | **Anxiety symptom**** | | |  | **Depressive symptom**** | | |  | **Any of the two**** | | | |
|  | Low  resilience | Moderate resilience | P value |  | Low  resilience | Moderate resilience | P value |  | Low  resilience | Moderate  resilience | P value |  |
| **Sex** |  |  | 0.818 |  |  |  | 0.266 |  |  |  | 0.267 |  |
| Male | 4.25 (0.65, 28.02) | 1.41 (0.21, 9.37) |  |  | 7.83 (1.19, 51.45) | 3.20 (0.49, 21.05) |  |  | 8.14 (1.25, 53.11) | 3.29 (0.50, 21.52) |  |  |
| Female | / | / |  |  | 4.57 (1.28, 16.30) | 2.49 (0.69, 8.97) |  |  | 5.00 (1.37, 18.31) | 2.66 (0.72, 9.82) |  |  |
| **Age (years)** |  |  | 0.786 |  |  |  | 0.333 |  |  |  | 0.440 |  |
| <30 | 1.81 (0.35, 9.25) | 0.52 (0.10, 2.88) |  |  | 3.54 (0.56, 22.36) | 1.02 (0.16, 6.54) |  |  | 3.75 (0.58, 24.47) | 1.21 (0.18, 7.95) |  |  |
| 30～39 | / | / |  |  | 4.25 (0.58, 24.47) | 1.65 (0.18, 7.95) |  |  | 4.37 (0.94, 20.35) | 1.64 (0.34, 7.90) |  |  |
| 40～49 | / | / |  |  | / | / |  |  | / | / |  |  |
| ≥50 | / | / |  |  | 6.36 (0.92, 44.07) | 3.69 (0.53, 25.73) |  |  | 6.77 (0.99, 46.44) | 3.80 (0.55, 26.24) |  |  |
| **Location** |  |  | 0.144 |  |  |  | 0.884 |  |  |  | 0.779 |  |
| Urban | 7.02 (1.01, 48.58) | 2.69 (0.39, 18.63) |  |  | 4.72 (1.61, 13.82) | 2.23 (0.76, 6.56) |  |  | 5.04 (1.72, 14.77) | 2.37 (0.80, 6.96) |  |  |
| Rural | / | / |  |  | / | / |  |  | / | / |  |  |
| **Education†** |  |  | 0.972 |  |  |  | 0.872 |  |  |  | 0.390 |  |
| High school and below | 5.50 (0.82, 37.00) | 1.60 (0.24, 10.86) |  |  | 2.95 (1.10, 7.92) | 1.42 (0.52, 3.84) |  |  | 3.21 (1.18, 8.70) | 1.49 (0.54, 4.08) |  |  |
| Bachelor degree | / | / |  |  | / | / |  |  | / | / |  |  |
| Master degree | / | / |  |  | / | / |  |  | / | / |  |  |
| **Marital status** |  |  | 0.371 |  |  |  | 0.018* |  |  |  | 0.018* |  |
| Unmarried | 4.71 (0.68, 32.50) | 1.81 (0.26, 12.57) |  |  | 9.49 (1.38, 65.26) | 3.63 (0.53, 25.12) |  |  | 10.11 (1.47, 69.53) | 3.96 (0.57, 27.39) |  |  |
| Married | / | / |  |  | 2.76 (0.82, 9.29) | 1.33 (0.39, 4.49) |  |  | 2.98 (0.87, 10.24) | 1.38 (0.40, 4.77) |  |  |
| Others | / | / |  |  | / | / |  |  | / | / |  |  |
| **Household size** |  |  | 0.561 |  |  |  | 0.715 |  |  |  | 0.670 |  |
| Solitary | / | / |  |  | / | / |  |  | / | / |  |  |
| 2 | / | / |  |  | 3.86 (0.80, 18.51) | 2.16 (0.45, 10.43) |  |  | 4.09 (0.81, 20.50) | 2.34 (0.46, 11.81) |  |  |
| 3 | 3.14 (0.59, 16.80) | 0.79 (0.14, 4.36) |  |  | 5.52 (0.83, 36.52) | 2.77 (0.43, 18.04) |  |  | 6.02 (0.89, 40.75) | 2.81 (0.42, 18.77) |  |  |
| ≥4 | / | / |  |  | 4.13 (0.58, 29.42) | 1.62 (0.22, 12.02) |  |  | 4.46 (0.62, 31.99) | 1.75 (0.24, 13.03) |  |  |
| **Housing** |  |  | 0.191 |  |  |  | 0.236 |  |  |  | 0.120 |  |
| Purchased | 6.77 (1.03, 44.31) | 2.06 (0.31, 13.59) |  |  | 11.73 (1.77, 77.53) | 5.07 (0.77, 33.55) |  |  | 12.95 (1.95, 85.93) | 5.34 (0.80, 35.45) |  |  |
| Rental | / | / |  |  | 3.07 (0.91, 10.36) | 1.64 (0.48, 5.61) |  |  | 3.08 (0.91, 10.44) | 1.70 (0.50, 5.80) |  |  |
| **Income§** |  |  | 0.860 |  |  |  | 0.011* |  |  |  | 0.012* |  |
| Lower level | 3.13 (0.48, 20.38) | 1.07 (0.16, 7.08) |  |  | 2.68 (0.86, 8.37) | 1.62 (0.51, 5.13) |  |  | 2.79 (0.88, 8.82) | 1.64 (0.51, 5.24) |  |  |
| Higher level | / | / |  |  | 12.62 (1.81, 88.10) | 4.83 (0.68, 34.13) |  |  | 13.34 (1.92, 92.95) | 5.07 (0.72, 35.74) |  |  |
| **Young children**†† |  |  | 0.644 |  |  |  | 0.495 |  |  |  | 0.542 |  |
| No | 7.28 (1.05, 50.54) | 2.39 (0.34, 16.68) |  |  | 4.46 (1.54, 12.91) | 2.02 (0.69, 5.88) |  |  | 4.82 (1.66, 14.00) | 2.17 (0.74, 6.33) |  |  |
| Yes | / | / |  |  | / | / |  |  | / | / |  |  |
| **Chronic disease** |  |  | 0.627 |  |  |  | 0.678 |  |  |  | 0.623 |  |
| No | 8.22 (1.27, 53.33) | 2.69 (0.41, 17.67) |  |  | 7.64 (1.94, 30.15) | 3.53 (0.89, 14.04) |  |  | 8.26 (2.10, 32.46) | 3.73 (0.94, 14.81) |  |  |
| Yes | / | / |  |  | 3.50 (1.06, 11.56) | 1.69 (0.51, 5.61) |  |  | 3.44 (1.04, 11.36) | 1.65 (0.50, 5.48) |  |  |
| **COVID-19 history** |  |  | 0.795 |  |  |  | 0.231 |  |  |  | 0.176 |  |
| No | 4.26 (0.65, 27.93) | 1.39 (0.21, 9.26) |  |  | 3.57 (1.05, 12.19) | 1.44 (0.42, 4.97) |  |  | 3.91 (1.12, 13.65) | 1.51 (0.43, 5.35) |  |  |
| Yes | / | / |  |  | 9.62 (1.34, 69.12) | 5.13 (0.71, 37.23) |  |  | 10.09 (1.41, 72.12) | 5.33 (0.74, 38.43) |  |  |
| * A P value for interaction less than 0.05 is considered to be statistically significant.  **†** Junior college in China was assigned to “high school and below” in our analyses. § We divided the income brackets based on the official average monthly income for China and Korea in 2022. Combining our questionnaire setup, we set income below 3000 RMB (China) and 3 million won (Korea) as the “Lower level”.  †† Young children were defined as children attending high school or younger.  ** The high resilience group were set as the reference group in all models. | | | | | | | | | | | | |
